# Prevalence and impact on the outcome of myosteatosis in patients with cirrhosis: a systematic review and meta-analysis

**DOI:** 10.1007/s12072-023-10632-8

**Published:** 2024-02-08

**Authors:** Aikaterini Kamiliou, Vasileios Lekakis, Lampros Chrysavgis, Evangelos Cholongitas

**Affiliations:** First Department of Internal Medicine, Laiko General Hospital, Medical School of National and Kapodistrian University of Athens, 17 Agiou Thoma Street, 11527 Athens, Greece

**Keywords:** Poor muscle quality, Cirrhosis, End stage liver disease, Chronic liver disease, Prognosis, Outcome, Non-alcoholic fatty liver disease, Child–Pugh, Hepatic encephalopathy, diabetes mellitus, Frailty

## Abstract

**Background:**

Myosteatosis in cirrhotic patients has been evaluated in limited studies with conflicting results and no systematic review or meta-analysis have been performed in this setting.

**Methods:**

We searched for all articles published until June 2023 to evaluate the prevalence of myosteatosis in cirrhosis and chronic liver disease.

**Results:**

Seventeen studies focused on cirrhosis and five studies in patients with chronic liver disease were included: the overall pooled prevalence of myosteatosis was 46% [95% Confidence Interval (CI) 36–57%] and 33% (95% CI 15–59%), respectively (*p* = 0.35). Among the studies with cirrhosis, the prevalence of myosteatosis was higher in those using the body mass index-based definition of myosteatosis (56%), than gender-based (36%) or other criteria (21%) (*p* < 0.01); was higher in women than in men (61% vs 45%), in Child–Pugh class C than A or B (57% vs 49% vs 50%), in non-alcoholic fatty liver disease (NAFLD)- than viral-associated cirrhosis (57% vs 43%), but these differences were not statistically significant (*p* > 0.05). Cirrhotic patients with myosteatosis, compared to those without myosteatosis, had more frequently a previous history of hepatic encephalopathy (32% vs 15%, *p* = 0.04), less frequently a previous history of variceal bleeding (46% vs 65%, *p* < 0.01), were more likely to suffer from diabetes mellitus (27% vs 18%, *p* < 0.01), while they had higher mortality rates (40% vs 14%, *p* = 0.02).

**Conclusion:**

Myosteatosis is highly prevalent in patients with cirrhosis, particularly in those with NAFLD-associated cirrhosis. Myosteatosis is associated with hepatic encephalopathy, while it seems to have a negative impact on the outcome.

**Supplementary Information:**

The online version contains supplementary material available at 10.1007/s12072-023-10632-8.

## Introduction

Although sarcopenia has been linked to reduced muscle quantity and low muscle quality determined by changes in muscle composition associated with pathological accumulation of collagen or lipids, it can also lead to poor muscle function and burdened physical performance [1]. Myosteatosis is defined as fat deposition into muscle and characterized as intermuscular adipose tissue (fat beneath the deep fascia), intramuscular adipose tissue (fat between and/or within myocytes), and/or intramyocellular lipids (i.e. as lipid droplets) [1, 2]. The existence of fat within the muscles can diminish muscle performance by disturbing the alignment of muscle fibers, thereby impairing their contractility and normal function [2]. Sarcopenia and myosteatosis often coexist in cirrhotic patients, but myosteatosis is considered a distinct entity that can be observed even in the absence of sarcopenia [2].

The pathogenesis of myosteatosis in cirrhosis has not been in depth elucidated, but hyperammonemia, which results in skeletal muscle ammonia uptake, has been suggested to promote skeletal muscle mitochondrial dysfunction, decreased lipid oxidation, and subsequent lipid deposition in muscles [1, 2]. In addition, systemic inflammation, and oxidative stress, which are commonly observed in liver cirrhosis, are associated with metabolic dysfunction in skeletal muscle, impaired muscle protein synthesis, turnover, and function [1, 2]. The diagnosis of myosteatosis is usually based on computed tomography (CT) or magnetic resonance imaging (MRI), which can distinguish fat from muscle by analysing tissue attenuation composition, which corresponds to the lipid content [3]. Myosteatosis can be detected by the reduced muscle attenuation at a specific cross sectional muscle area, usually at the level of third lumbar vertebra (L3) [1]. Although CT has several limitations, it is the most used tool to evaluate myosteatosis, particularly in cirrhotic patients, as it is usually available as part of standard of care [4]. However, unlike sarcopenia, no standardized criteria regarding the optimal modality and specific cut offs for the diagnosis of myosteatosis have been established [1, 2].

Although it is estimated that myosteatosis is highly prevalent in patients with cirrhosis [1, 2], that assumption has been evaluated in limited individual studies with inconsistent results. Thus, our aim was to perform a comprehensive systematic review and meta-analysis to assess the prevalence of myosteatosis in patients with chronic liver disease and cirrhosis overall as well as in specific different subgroups and to evaluate its prognostic impact on patients with end stage liver disease.

## Methods

### Data sources and searches

Medline/PubMed, Embase and Cochrane databases were searched for studies published until June 2023 according to the Preferred Reporting Items for Systematic Reviews and Meta-Analyses (PRISMA) to identify all medical literature included under the keywords “myosteatosis” or “muscle quality” or “muscle alterations” AND “cirrhosis” OR “cirrhotic patients” OR “liver cirrhosis” OR “end stage liver disease” OR “chronic liver disease” OR “liver disease”. In addition, we searched all relevant review articles to identify further original studies as well as we searched the abstracts from the major Hepatology and Liver Transplant congresses during the last year. Finally, we scrutinized the references of each article for additional potential eligible studies.

### Study selection

All studies published in English language were considered eligible if they fulfilled all the following criteria: (1) they were randomised controlled trials or observational cohort studies, (2) they included adult patients (> 18 years) with chronic liver disease or cirrhosis, (3) the definition of myosteatosis was provided, and (4) the prevalence of myosteatosis was reported. Literature search for relevant studies was performed independently by two reviewers (AK, VL) who determined which studies could be potentially included after having screened titles and abstracts. Each study in the list of the preselected papers was evaluated by two independent reviewers (EC, LC) to determine whether it fulfilled all the inclusion criteria. Exclusion criteria were case reports and review articles as well as studies including patients < 18 years old or patients suffering from non-liver diseases.

### Data extraction and quality assessment

Data extraction from the finally selected papers was performed by two authors (AK, LC) according to a predefined form, while any disagreement was resolved by discussion with another author (EC). Data extracted for selected studies included the first author, date of publication, country of origin and centre(s), type of study, sample size, source of cirrhotic patients [candidates on the waiting list for liver transplantation (LT) or not], as well as gender, mean or median age. Moreover, we searched for the definition of myosteatosis and the method for its evaluation recording the specific cut offs which are used to define myosteatosis. In addition, etiology of chronic liver disease and cirrhosis [viral, non-alcoholic fatty liver disease (NAFLD), alcoholic liver disease (ALD) or other], severity of liver disease based on Child–Pugh (CP) and Model for End-Stage Liver Disease (MELD) scores (mean or median), previous history of hepatic encephalopathy or other liver-related complications, the number of patients with hepatocellular carcinoma (HCC) and type II diabetes mellitus (T2DM) and laboratory values namely mean or median serum creatinine, bilirubin and INR, were also evaluated. The same data were extracted from the patients with or without myosteatosis, whenever available. Finally, mortality or survival rates were also recorded, without considering post-LT outcomes.

### Data synthesis and analysis

We used a descriptive approach to summarize study characteristics and outcomes with regard to the presence of myosteastosis. Quantitative variables were expressed as mean values ± standard deviation and/or median values along with the corresponding ranges. Level of significance was set to 0.05, thus tests with p-values less than 0.05 were considered as statistically significant.

Meta-analysis was performed using a generalized linear mixed model (GLMM) [5]. The two-sided confidence intervals for the single proportions of each individual study were computed using the Clopper and Pearson method [6]. The between-study variance component (τ2) was estimated applying the maximum likelihood method, based on marginal distribution [7]. I2 was used to measure heterogeneity, and I2value of 25%, 50% and 75% represented low, moderate, and high degrees of heterogeneity, respectively. Random effects or fixed effects models were used depending on the presence of substantial heterogeneity across trials respectively [8]. The pooled proportions along with the 95% confidence intervals (CI) and the prediction intervals (PI) were calculated [9]. Analysis was conducted in R v4.1.2 using meta-packages and metaprop functions [10].

## Results

### Studies focused on cirrhosis

In total, 62 articles were initially identified from the literature search, but only 23 studies fulfilled the inclusion criteria and underwent further evaluation (Suppl. Figure S1) [11–33]. Two studies from a single center in the Netherlands [23, 28] as well as three studies from a single center in China (17,31,33) had overlapping study periods, and therefore only the most recent studies [17, 23] were included. Similarly, three studies from a single center in Canada [12, 29, 30] had overlapping study periods, but the oldest one [12] which provided additional data on patients with myosteatosis was included. Finally, four studies [11, 18, 20, 32] were from the same single center in Italy but two [18, 32] of them had overlapping study periods and we included the newest study [18]. Thus, 17 studies [11–27], that evaluated the prevalence of myosteatosis in cirrhotic patients, fulfilled all inclusion criteria and were included in the final analysis. Four studies were derived from Italy [11, 18, 20, 22], three from China [17, 19, 21], two from Japan [14, 15], as well as two from Germany [16, 25] and the USA [24, 27] and one from Canada [12], Greece [13], South Africa [26] and the Netherlands [23]. In only one study [24] MRI was used for the evaluation of myosteatosis. Ten of the 17 studies had a retrospective design [11, 14–17, 19, 22–24, 26]. The Newcastle–Ottawa scale (NOS), was used to assess the quality of the included studies [34]. Based on that, the studies had low risk of bias (NOS scored > 5) (Suppl. Table 1).

### Studies in patients with chronic liver disease

In total, 106 studies were initially identified regarding myosteatosis in patients with chronic liver disease, but only 6 studies met the inclusion criteria [35–40] (Suppl. Figure S1). Two studies [38, 40] from a single center in Japan had overlapping study periods, and we included the study with the largest cohort [38]. Thus, 5 studies were finally included [35–39]. In 4 of them [36–39], the presence of cirrhosis was not an exclusion criterion, but the proportion of patients with cirrhosis was relatively small whenever this was available (Suppl. Table 2). Nevertheless, no separate data were provided for patients with or without cirrhosis in these studies. The included studies were derived from different countries (Korea [35], Taiwan [36], Sweden [37], Japan [38] and Germany [39]), while different definitions were used for myosteatosis (Suppl. Table 2).

### Characteristics of patients

#### Studies focused on cirrhosis

In total, 4136 cirrhotic patients [mean age: 60.2 years, 64.5% (2674/4136) males] were evaluated. In the majority of patients (56.3% or 2327/4136) the diagnosis of myosteatosis was based on muscle/m^2^ radiodensity at L3 < 41 HU for patients with dry body mass index (BMI) < 25 kg/m^2^ and < 33 HU for those with ΒΜΙ ≥ 25 kg/m^2^ (i.e. BMI-based definition) [11–15, 18–20, 25–27]. In 1368 (33%) patients the gender-based definition using different cut offs between males and females (e.g. < 26.6 HU in females and < 28.6 HU in males) [16, 17, 21, 22] was used for diagnosis of myosteatosis, while in 441 (10.7%) patients various other criteria were applied [23, 24]. According to the available data, mean CP and MELD scores were 7.2 and 13.8, respectively. Chronic viral hepatitis (B or C) was the underlying cause of cirrhosis in 39% (n = 1615) of patients, while HCC was present in 39.7% (870/2191) of patients [11, 12, 14, 16, 22, 25–27] and 28.8% of the patients (668/2316) had a previous history of hepatic encephalopathy [11, 13, 14, 17, 18, 20–22, 24, 27]. In addition, 32.7% (1355/4136) of patients were from Asia [14, 17, 19, 21] and 1328 patients were candidates for LT during the follow-up period. Finally, among the 1631 patients with available data, 704 (43%), 717 (44%) and 210 (13%) of them were classified as CP class A, B and C, respectively [11, 14, 15, 17, 19, 20, 22, 27].

##### Characteristics of patients with myosteatosis

In total, 1768 cirrhotic patients (mean age: 64.6 years, 58.7% (718/1223 males) had myosteatosis. According to the available data, CP and MELD scores were 8.7 and 13.6, respectively. Chronic viral hepatitis (B or C) was the underlying cause of chronic liver disease in 38.5% (521/1358) of patients, HCC was present in 55.5% (260/468) of patients [12, 22], while 30.8% (182/591) [11, 13, 17, 18, 20, 21] and 46% (106/230) [17, 21] had a previous history of hepatic encephalopathy and variceal bleeding, respectively. In addition, 26% (n = 459) patients were from Asia and 480 (27.1%) patients were candidates for LT. Finally, among the 961 patients with available data, 357 (37%), 394 (41%) and 210 (22%) of them were classified as CP class A, B and C, respectively [11, 12, 14, 15, 17, 18, 20, 22] (Suppl. Table 1).

##### Characteristics of patients without myosteatosis

In total, 2368 cirrhotic patients had no myosteatosis (mean age 55.5 years, 64% or 949/1484 males, CP score: 6.1 and MELD score: 12.8). Chronic viral hepatitis (B or C) was the underlying cause of chronic liver disease in 47% (765/1628) of patients, 50.4% (182/361) of patients had HCC (12,22) whereas a previous history of hepatic encephalopathy and variceal bleeding was recorded in 13.2% (130/986) [11, 13, 17, 18, 20, 21] and 65% (470/723) [17, 21] of patients, respectively. In addition, 37.8% (896/2368) of patients were from Asia, while among the 737 patients with available data, 305 (41%), 360 (49%) and 72 (10%) were classified as CP class A, B and C, respectively [11, 14, 15, 17, 18, 20, 22] (Suppl. Table 1).

#### Studies in patients with chronic liver disease

In total, 4364 patients [mean age: 55.3 years, 2049 (47%) males] were evaluated [35–39]. Three studies [35–37] were focused on NAFLD including 3886 patients, one study [38] evaluated patients with various etiology of liver disease (n = 362) and one study [39] included patients with primary sclerosing cholangitis (n = 116) (Suppl. Table 2). In addition, 69.7% (3044/4364) of patients were from Asia. In total, 1421 patients had myosteatosis (mean age: 60.8 years, 42.6% (192/450 males), while 2943 had no myosteatosis (mean age: 48.7 years, 58.8% (323/549 males) (Suppl. Table 2).

### Prevalence of myosteatosis in total and in specific sub-groups

#### Studies focused on cirrhosis

**Fig. 1:**
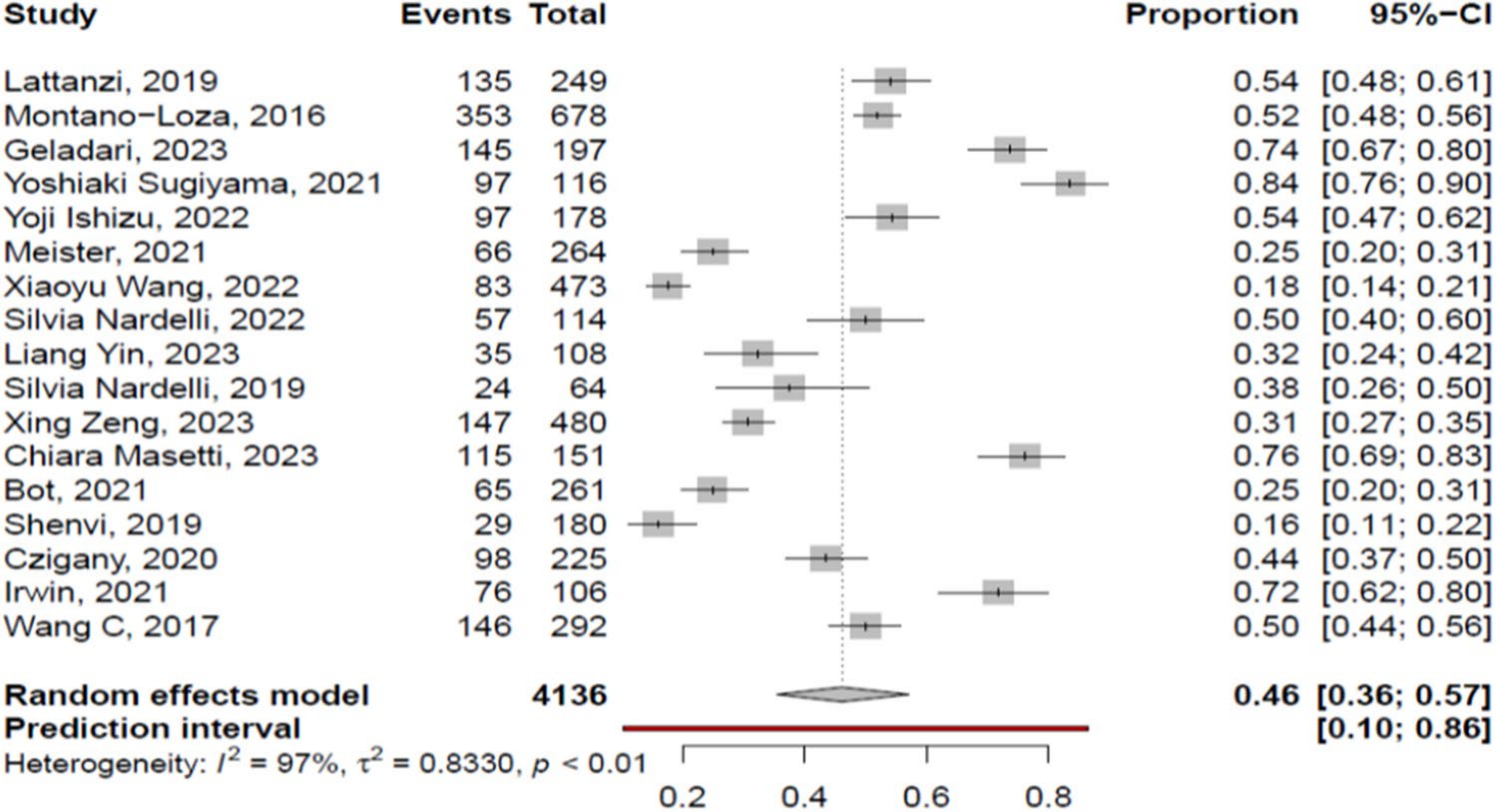
The pooled overall prevalence of myosteatosis in patients with cirrhosis in the included studies

**Fig. 2:**
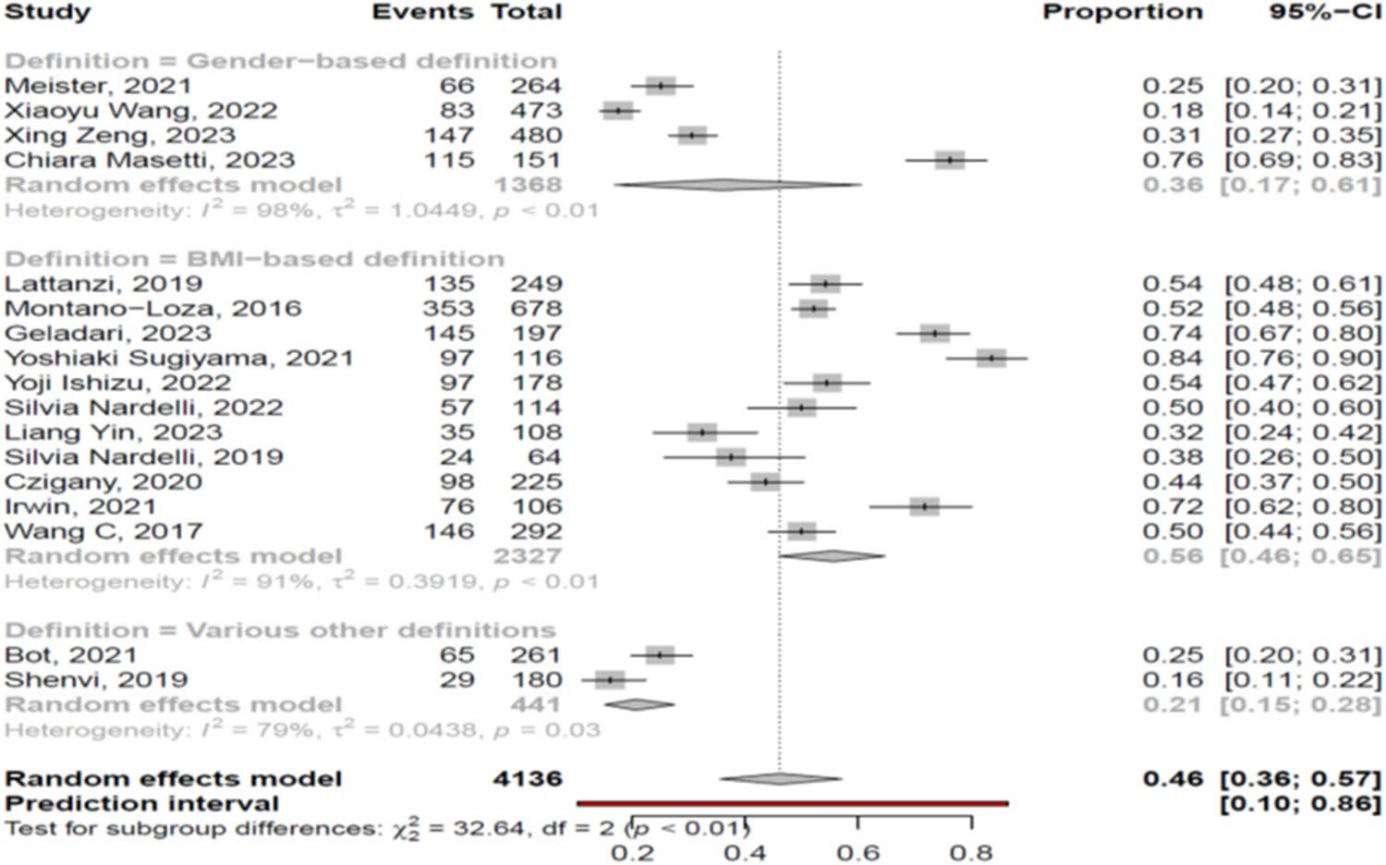
Forest plot of studies comparing the prevalence of myosteatosis according to the definition criteria. BMI: body mass index

**Fig. 3:**
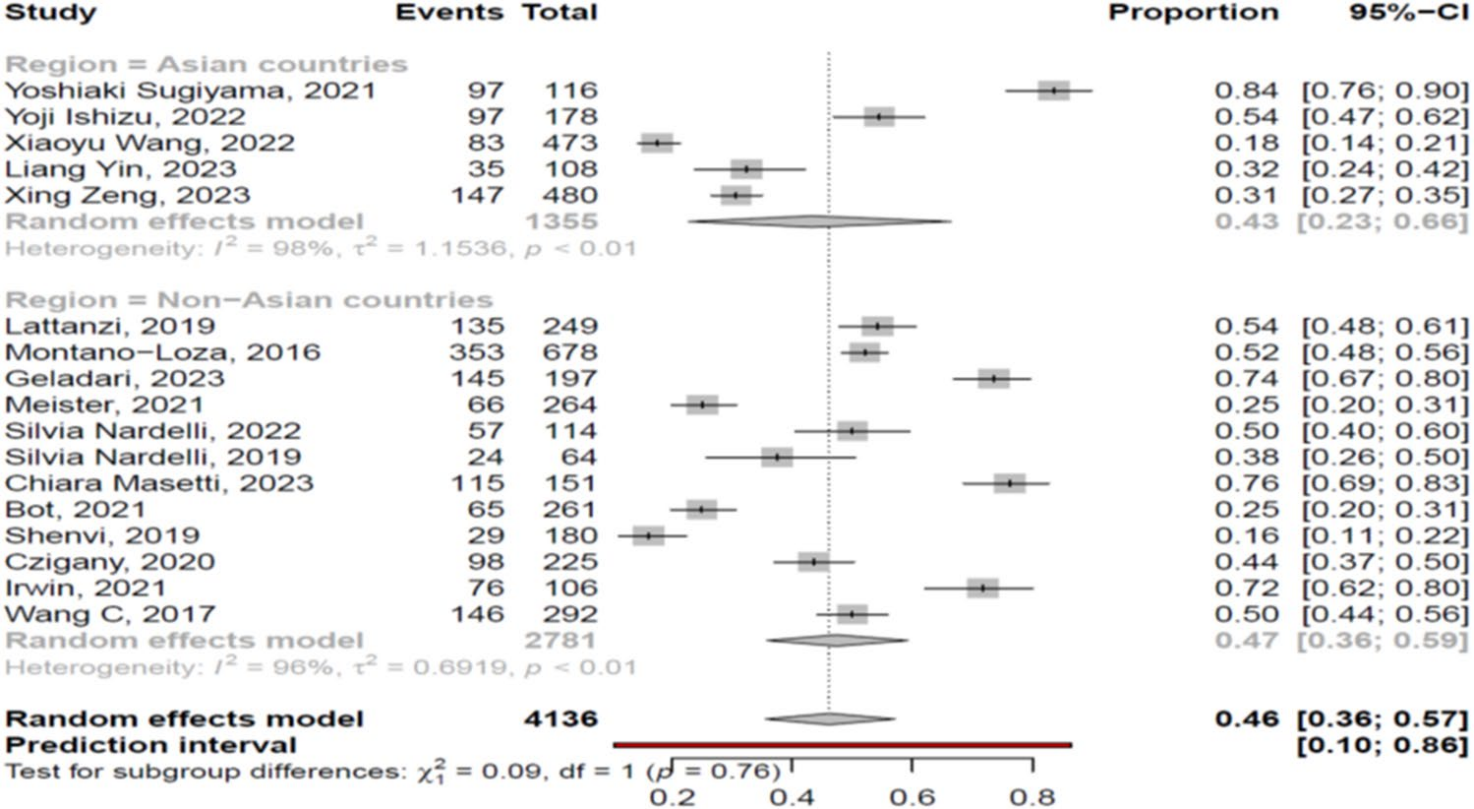
Forest plot of studies comparing the prevalence of myosteatosis according to the region of the studies (Asian vs non-Asian countries)

The overall pooled prevalence of myosteatosis in cirrhotic patients was 46% (95% CI 36–57%; heterogeneity, *p* < 0.01, primary study range 16–84%) (Fig. 1) [11–27] with no difference between prospective and retrospective studies [48% (95% CI 39–58%; heterogeneity, *p* < 0.01) vs 45% (95% CI 29–62%; heterogeneity, *p* < 0.01), respectively, p = 0.72]. Τhe pooled prevalence of myosteatosis was 56% (95% CI 46–65%; heterogeneity, *p* < 0.01), 36% (95% CI 17–61%; heterogeneity, *p* < 0.01) and 21% (95% CI 15–28%; heterogeneity, *p* = 0.03) in studies using the BMI-based definition, gender-based definition and various other criteria for the diagnosis of myosteatosis, respectively (*p* < 0.01) (Fig. 2). However, no significant difference in the pooled prevalence of myosteatosis was found between studies from Asia in comparison with non-Asian countries [43% (95% CI 23–66%; heterogeneity, *p* < 0.01) vs 47% (95% CI 36–59%; heterogeneity, *p* < 0.01), respectively, *p* = 0.76] (Fig. 3), regardless of definition criteria.

##### Gender

The pooled prevalence of myosteatosis was similar between men and women [45% (95% CI 31–60%; heterogeneity, *p* < 0.01) vs 61% (95% CI 42–77%; heterogeneity, *p* < 0.01), *p* = 0.20] (Suppl. Figure S2), regardless of definition criteria or geographical area. Nonetheless, in studies which used the BMI-based definition of myosteatosis, the prevalence of myosteatosis was significantly lower in men than women [51% (95% CI 40–62%; heterogeneity, *p* < 0.01) vs 73% (95% CI 59–84%; heterogeneity, *p* < 0.01), *p* = 0.02].

##### Severity of liver disease

**Fig. 4:**
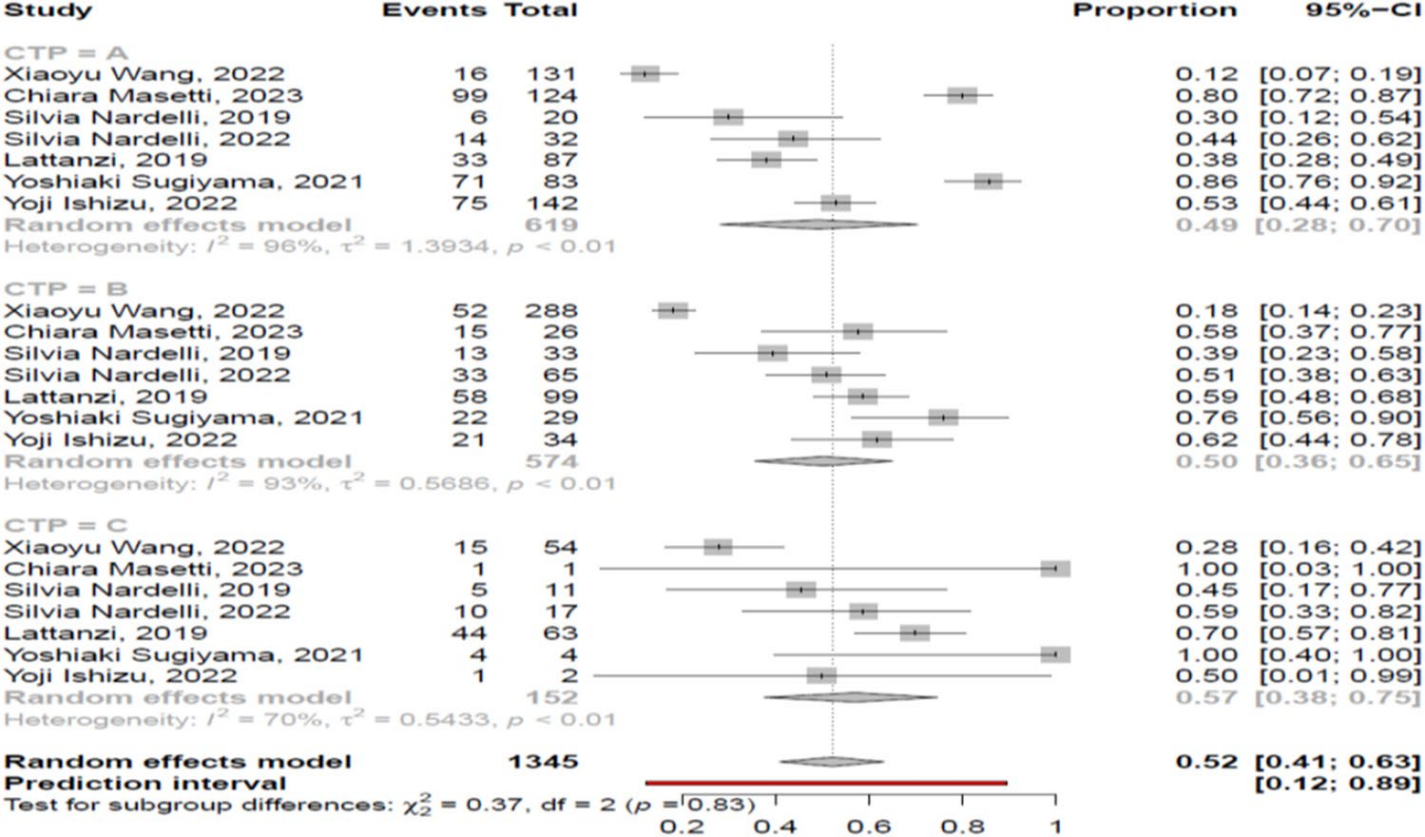
Forest plot of studies comparing the prevalence of myosteatosis according to the Child–Pugh classification

Based on the available data, myosteatosis was more frequent in patients with more severe liver disease, since the prevalence of myosteatosis was 49% (95% CI 28–70%; heterogeneity, *p* < 0.01), 50% (95% CI 36–65%; heterogeneity, *p* < 0.01) and 57% (95% CI 38–75%; heterogeneity, *p* < 0.01) in patients at CP class A, B and C, respectively. However, those differences were not statistically significant (*p* = 0.83) (Fig. 4). These findings were similar when BMI-based definition was used: 52% (95% CI 33–71%), 57% (95% CI 48–65%) and 66% (95% CI 56–75%), *p* = 0.28, respectively.

##### Etiology of liver disease

**Fig. 5:**
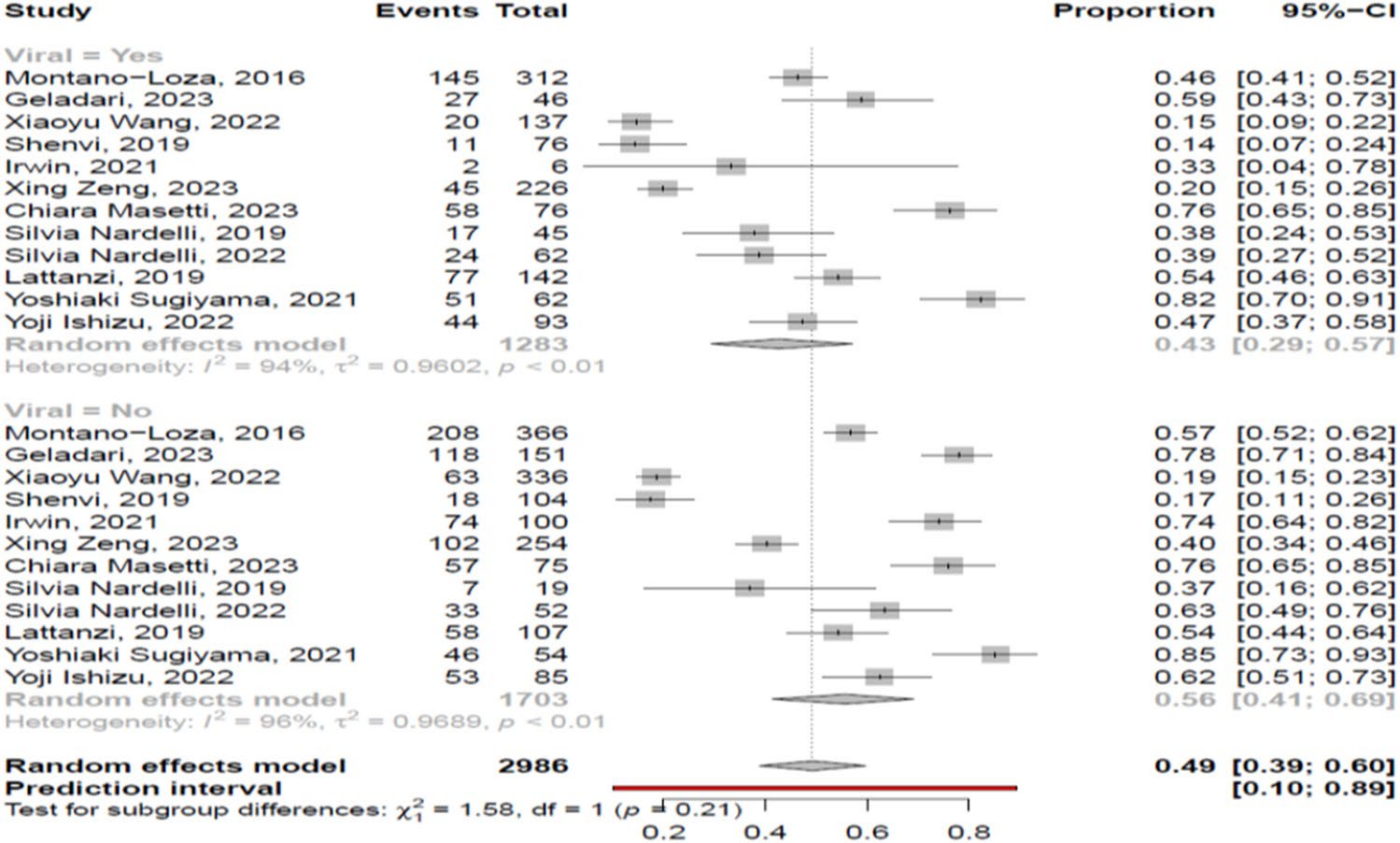
Forest plot of studies comparing the prevalence of myosteatosis according to the aetiology of underlying liver disease (viral vs non-viral)

The pooled prevalence of myosteatosis was lower in patients with viral-associated cirrhosis, compared to those with non-viral associated cirrhosis [43% (95% CI 29–57%; heterogeneity, *p* < 0.01) vs 56% (95% CI 41–69%; heterogeneity, *p* < 0.01)], but this difference was not significant (*p* = 0.21) (Fig. 5). These findings were similar irrespectively of definition criteria [e.g. BMI-based definition: 52% (95% CI 41–62%) vs 66% (95% CI 56–74%), respectively, *p* = 0.10; gender-based definition: 34% (95% CI 10–69%) vs 44% (95% CI 19–72%), respectively, *p* = 0.67]. Interestingly, the pooled prevalence of myosteatosis was similar between NAFLD- and ALD-associated cirrhotic patients [57% (95% CI 35–76%) vs 53% (95% CI 39–67%), *p* = 0.80].

#### Studies in patients with chronic liver disease

The overall pooled prevalence of myosteatosis was 33% (95% CI 15–59%; heterogeneity, *p* < 0.01, primary study range 11–82%), which was lower, compared to the overall pooled prevalence of myosteatosis in patients with cirrhosis (Suppl. Figure S3). However, this difference was not significant (*p* = 0.35).

##### Gender

The pooled prevalence of myosteatosis was lower in men than in women [29% (95% CI 0.04–79%; heterogeneity, *p* < 0.01) vs 45% (95% CI 0.17–76%; heterogeneity, *p* < 0.01)], but this difference was not significant (*p* = 0.61).

Based on the available data no other sub-group analysis could be performed.

### Characteristics of patients with and without myosteatosis

#### Studies focused on cirrhosis

##### History of cirrhosis-related complications

**Fig. 6:**
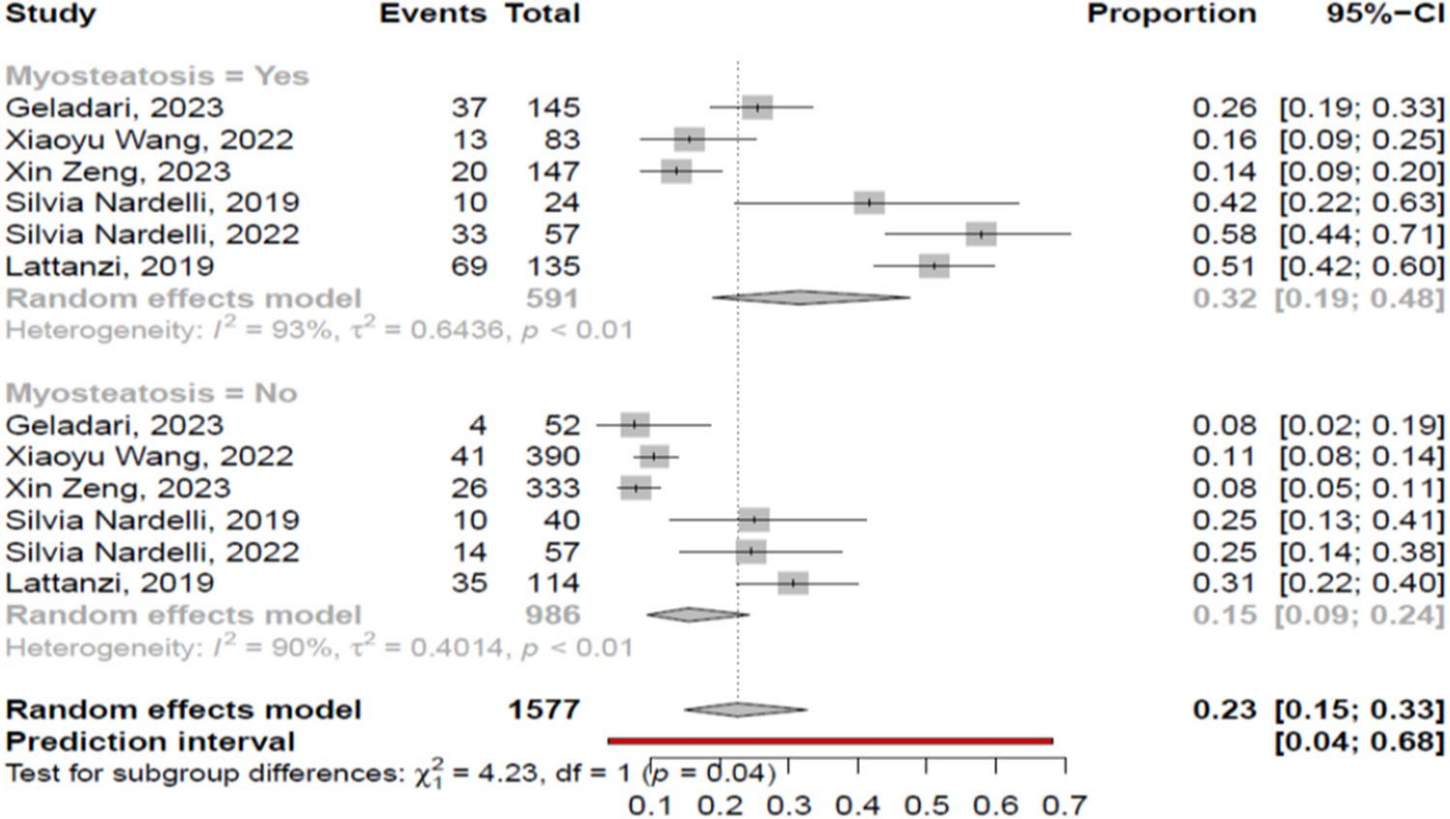
Forest plot of studies comparing the prevalence of previous history of hepatic encephalopathy between the patients with or without myosteatosis

The patients with myosteatosis, compared to those without myosteatosis, had significantly more frequently a previous history of hepatic encephalopathy [32% (95% CI 19–48%; heterogeneity, *p* < 0.01) vs 15% (95% CI 9–24%; heterogeneity, *p* < 0.01), *p* = 0.04] (Fig. 6). These findings were similar irrespectively of definition criteria [e.g. BMI-based definition: 43% (95% CI 30–57%) vs 21% (95% CI 13–32%),respectively, *p* = 0.01; gender-based definition: 14% (95% CI 10–19%) vs 9% (95% CI 7–12%), respectively, *p* = 0.03]. Concurrently, the former had significantly less frequently a previous history of variceal bleeding [46% (95% CI 40–53%; heterogeneity, *p* = 0.19) vs 65% (95% CI 61–68%; heterogeneity, *p* = 0.19), *p* < 0.01]. In addition, the patients with myosteatosis, compared to those without, had similar prevalence of ascites [62% (95% CI 41–79%; heterogeneity, *p* < 0.01) vs 53% (95% CI 38–67%; heterogeneity, *p* < 0.01), *p* = 0.48], as well as a previous history of severe infections/sepsis/spontaneous bacterial peritonitis (SBP) [10% (95% CI 7–15%; heterogeneity, *p* = 0.14) vs 6% (95% CI 2–19%; heterogeneity, *p* < 0.01), *p* = 0.41].

##### T2DM comorbidity

**Fig. 7:**
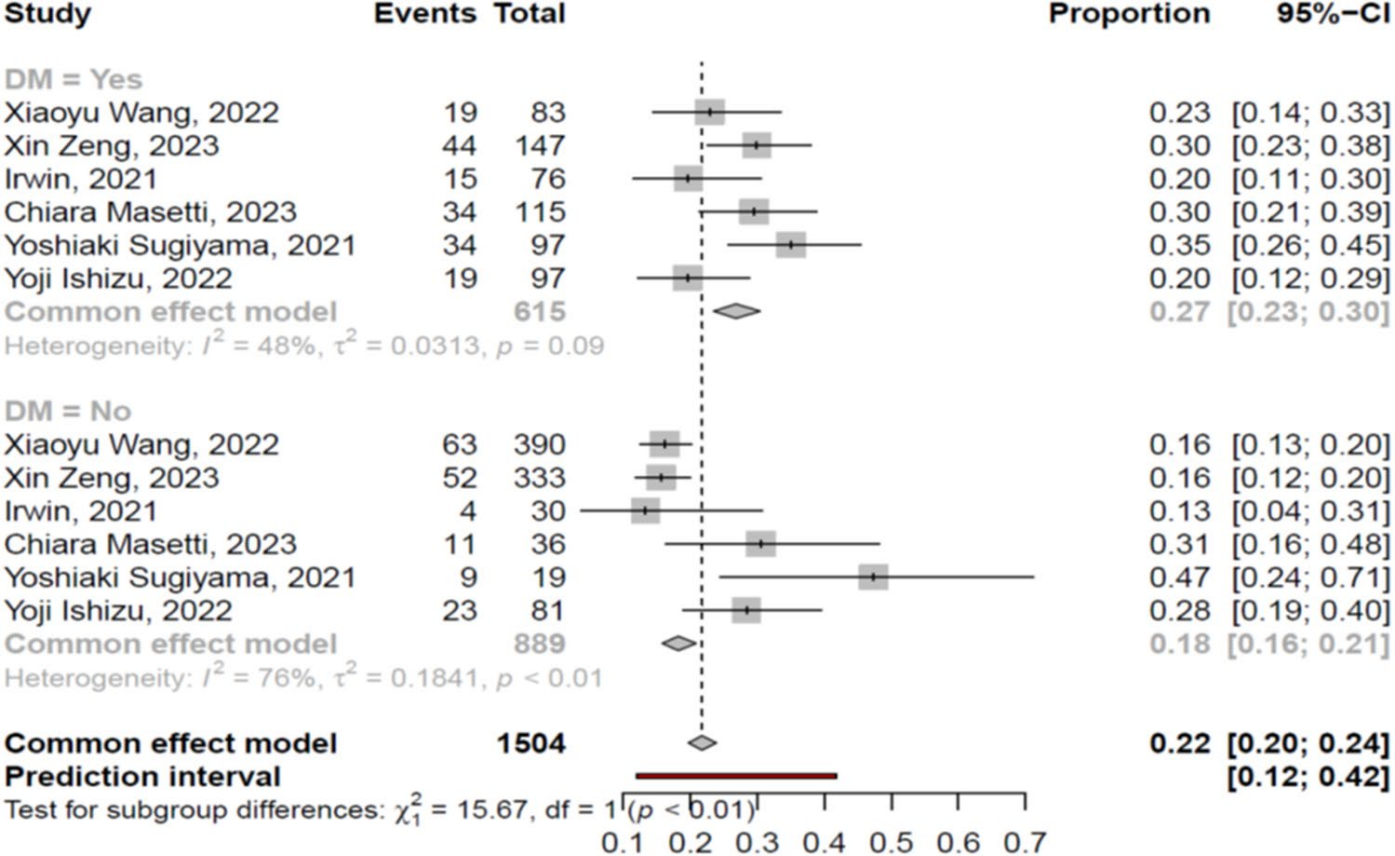
Forest plot of studies comparing the prevalence of diabetes mellitus between the patients with or without myosteatosis. DM: diabetes mellitus

The cirrhotic patients with myosteatosis suffered more commonly from T2DM, compared to those without myosteatosis [27% (95% CI 23–30%%; heterogeneity, *p* = 0.09) vs 18% (95% CI 16–21%; heterogeneity, *p* < 0.01), *p* < 0.01] (Fig. 7). These findings were confirmed when we evaluated only the studies used the gender-based definition [28% (95% CI 24–33%) vs 17% (95% CI 14–19%), respectively, *p* < 0.01], but not when we analyzed the studies used the BMI-based definition [25% (95% CI 17–34%) vs 28% (95% CI 16–43%), respectively, *p* = 0.71].

##### Outcome of patients with and without myosteatosis

**Fig. 8:**
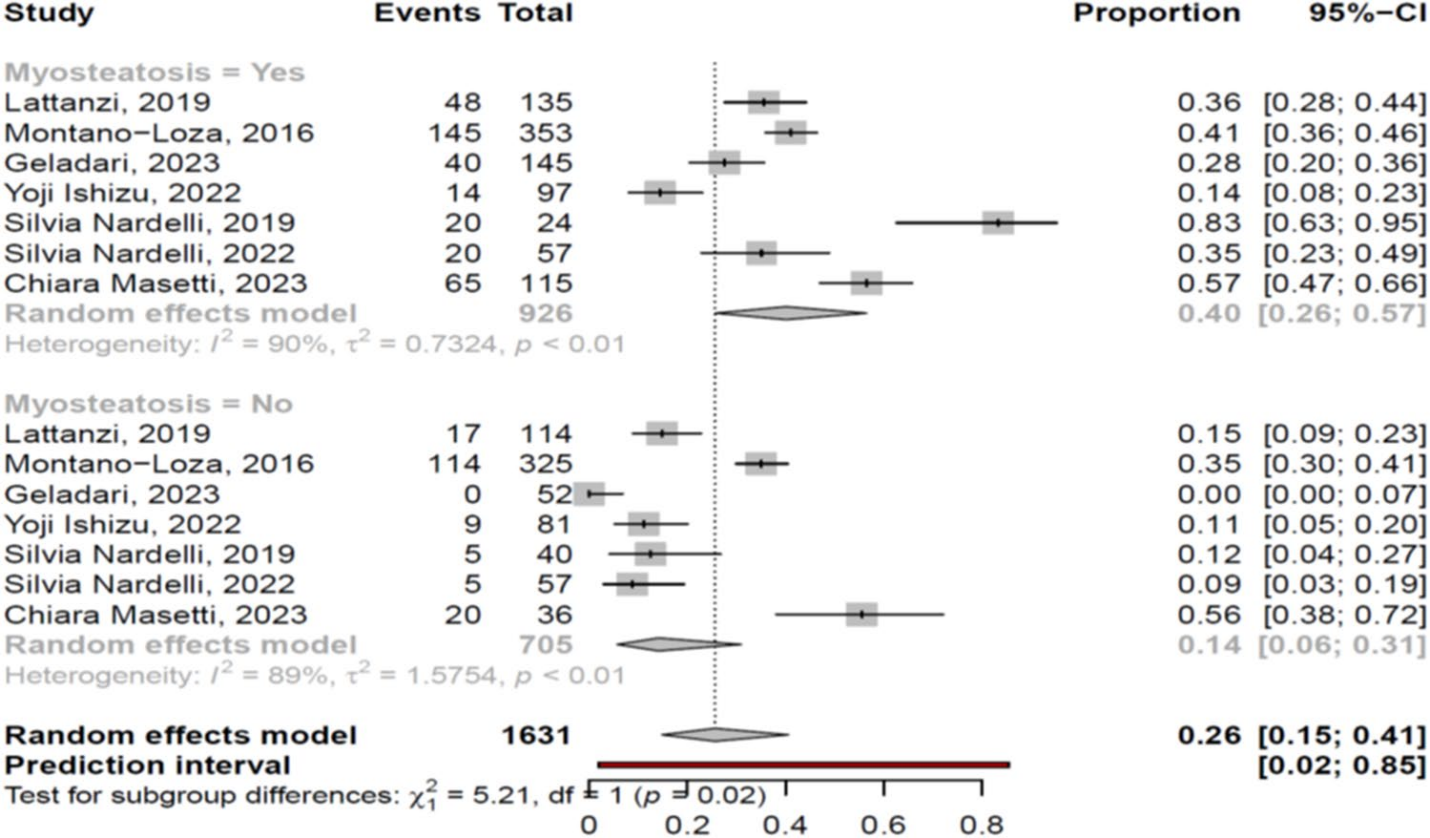
Forest plot for assessment of association between myosteatosis and mortality

The patients with myosteatosis had significantly lower survival than those without myosteatosis [pooled mortality rate: 40% (95% CI 26–57%; heterogeneity, *p* < 0.01) vs 14% (95% CI 6–31%; heterogeneity, *p* < 0.01), *p* = 0.02] (Fig. 8). These findings were confirmed when we evaluated the studies used the BMI-based definition: 37% (95% CI 22–56%) vs 11% (95% CI 5–23%), respectively, *p* < 0.01 (only one study used the gender-based definition). Among the studies that evaluated myosteatosis in cirrhotic patients [11–27], only 2 studies provided data regarding the causes of death: Geladari et al. [13] recorded only liver-related deaths in patients with myosteatosis, while in the study by Montano-Loza et al. [12] more frequently sepsis-related deaths and less frequently liver-related deaths were recorded in patients with myosteatosis in comparison to those without (Suppl. Table 1).

#### Studies in patients with chronic liver disease

Among the included studies, there were not available data regarding the characteristics of patients with and without myosteatosis, as well as data regarding mortality. Only one [38] of the included studies reported that the patients with myosteatosis had higher risk for HCC development, compared to those without.

### Publication bias

In order to evaluate the existence of publication bias, a funnel plot asymmetry test and Egger’s test were performed [41] (Suppl. Figure S4). No substantial asymmetry was revealed, as evidenced by the non-significant Egger’s test for a regression intercept (β_0_: 2.79; 95% CI -0.63–0.61; t:0.64; *p* = 0.53). The significant variation in findings across individual studies was addressed by employing the random effect model for all calculations and by conducting subgroup analyses based on specific criteria, including the different myosteatosis definitions, the country where the studies were conducted, the gender of patients as well as the severity and etiology of liver disease.

## Discussion

Dealing with the current literature, previous systematic reviews/meta-analyses have evaluated myosteatosis only in patients with various types of cancer (e.g. lung, gynecological and gastrointestinal) indicating a high prevalence and a negative prognostic impact on survival of those patients [42]. To the best of our knowledge, the present study is the first systematic review/meta-analysis that evaluated the prevalence of myosteatosis in patients with cirrhosis (overall and in several sub-groups), as well as its impact on mortality. Based on the current literature which included 17 relevant studies, we showed that almost half of cirrhotic patients have myosteatosis (pooled prevalence 46%, 95% CI 36–57%) (Fig. 1) [11–27], confirming that excessive fat infiltration in skeletal muscles is widely prevalent in patients with end stage liver disease [2]. In addition, the pooled prevalence of myosteatosis in studies with chronic liver disease was lower [33% (95% CI 15–59%]. However, this difference was not significant (*p* = 0.35), presumably because the studies with chronic liver disease included patients with cirrhosis, while NAFLD was the main etiology of the underlying liver disease. Interestingly, based on the literature data [35, 43, 44], the prevalence of myosteatosis in healthy adult subjects is reported to range from 10 to 25%, thus lower compared to patients with chronic liver disease/cirrhosis indicating that specific mechanisms are involved in the pathogenesis of myosteatosis in the setting of chronic liver disease and/or cirrhosis.

To date, there is no commonly accepted definition of myosteatosis which complicates the interpretation of literature findings. Nevertheless, most of the included studies in the present meta-analysis used the BMI-based criteria and fewer the gender-based or other criteria (Suppl. Table 1). However, it is considered that the BMI-based proposed cut offs may not be appropriate in cirrhotic patients with ascites since the latter often suffer from high fluid retention and as a result gender-based criteria may be more suitable due to the higher lipid storage capacity in females, compared to males [2]. Interestingly, in this meta-analysis, the reported prevalence of myosteatosis was significantly higher in studies using the BMI-based criteria (56% (95% CI 46–65%), compared to those using the gender-based or other criteria [36% (95% CI 17–61%) and 21% (95% CI 15–28%), respectively] (*p* < 0.01) (Fig. 2), indicating that a consensus is needed in order to define the optimal criteria for the assessment of myosteatosis in the context of cirrhosis. Interestingly, no difference in the prevalence of myosteatosis was found between Asian and non-Asian studies [43% vs 47%, *p* = 0.76] (Fig. 3). However, it should be mentioned that Asian and non-Asian studies used the same cut offs, although it is accepted that Asians harbor more amount of body fat at the same BMI, compared to other ethnic populations. In addition, the prevalence of myosteatosis was similar between men and women [45% vs 61%, *p* = 0.20], but when we assessed only the studies which used the BMI-based criteria, the prevalence was higher in women, compared to men [73% vs 51%, *p* = 0.02].

In our meta-analysis we showed that myosteatosis was more prevalent in patients with CP class C than CP class A or B, although that difference was not significant (57% vs 49% and 50%, respectively, *p* = 0.83) (Fig. 4). Τhis finding was based on seven studies, and it is in accordance with the study by Geladari et al. [13], who found that cirrhotic patients with myosteatosis, compared to those without, had significantly higher CP score (median 8 vs 5, *p* < 0.001). Similarly, Montano-Loza et al. [12] found that patients with myosteatosis had higher CP score, compared to patients with no muscular abnormalities (mean 10 vs 8, *p* < 0.001). However, both studies were not included in our analysis due to unavailable data regarding the number of patients with and without myosteatosis based on CP classification (CP class A, B or C). It is considered that myosteatosis precedes subsequent muscle wasting and sarcopenia and it seems to occur when excess fat is accumulated in muscle tissue which serves as an ectopic lipid storage in patients with obesity or increased total body fat [13, 15, 17, 38], i.e. in cirrhotic patients who are at early stages with relatively preserved liver function. However, hyperammonemia, hyper-endotoxemia and malnutrition, which are more prominent in advanced liver disease, have been associated with the development of myosteatosis [1, 2]. Nevertheless, further studies are needed to elucidate better this issue.

Literature data have shown that myosteatosis is commonly observed in patients with metabolic syndrome, T2DM, and NAFLD without cirrhosis, indicating the close relationship between myosteatosis and insulin resistance [37]. Thus, as may be expected, in our meta-analysis we confirmed these findings in the setting of end stage liver disease, since the prevalence of myosteatosis was higher in cirrhotic patients with than those without T2DM (27% vs 18%, *p* < 0.01) (Fig. 7), but this was confirmed only when we evaluated the studies used the gender-based definition (28% vs 17%, *p* < 0.01). In addition, we highlighted that the etiology of the underlying liver disease was correlated with the presence of myosteatosis, as the latter was more prevalent in NAFLD-associated cirrhotic patients, compared to their ALD- or viral-associated cirrhotic counterparts (57% vs 53% vs 43%, respectively). However, these differences were not statistically significant.

As in sarcopenia [2], recent studies have also shown that myosteatosis may be a risk factor for the development of hepatic encephalopathy, which is mediated by the reduction of skeletal muscle capacity to remove ammonia or via myosteatosis-induced inflammatory state which increases the ammonia toxicity [1, 2]. In addition, lower number of hepatic encephalopathy episodes were reported in patients with an improvement of nutritional status and reduction in fat mass after trans jugular intrahepatic portosystemic shunt compared to those without improvement in nutritional status [19]. Our meta-analysis confirmed the association between hepatic encephalopathy and myosteatosis, as the patients with myosteatosis had more frequently a previous history of hepatic encephalopathy, compared to those without [32% vs 15%, respectively, *p* = 0.04] (Fig. 6) irrespectively of definition criteria, while they had less frequently a previous episode(s) of variceal bleeding (46% vs 65%, *p* < 0.01). However, no clear explanation can be provided for the latter finding. Finally, no difference was found concerning the prevalence of ascites and infections/SBP between the patients with and without myosteatosis.

Myosteatosis has been related to higher risk of adverse outcome in several specific populations, including patients with malignancies [42] and those under hemodialysis [45]. In cirrhotic patients, it has been found that both myosteatosis and sarcopenia were independent predictors of mortality [11], while a new prognostic score including MELD, sarcopenia and myosteatosis has been proposed [11, 21]. Although this new score is based on objective variables [11], further validation is needed. In our meta-analysis, we found that cirrhotic patients with myosteatosis had worse survival, compared to those without myosteatosis (40% vs 14%, *p* = 0.02) (Fig. 8) (studies used the BMI-based definition: 37% vs 11%, *p* < 0.01; only one study used gender-based definition). This finding indicated that the quality of skeletal muscles may represent an additional important prognostic factor in patients with cirrhosis presumably related to the presence of poor quality of life, frailty, malnutrition and deterioration of portal hypertension. However, it should be mentioned that no conclusions could be drawn regarding the causes of death between cirrhotic patients with and without myosteatosis, thus further studies are needed to clarify better this issue. In addition, it would be interesting to investigate the association between myosteatosis and muscle function, but only four studies [13, 14, 22, 27] provided relevant data. Although a positive correlation was found between myosteatosis and functional capacity in these studies, meta-analysis could not be performed since different tools were used to assess functional capacity (e.g. physical performance battery test, handgrip strength).

This meta-analysis has some limitations, including the fact that although the quality of the studies was high (all NOS > 5), 10 of the 17 studies were retrospective leading to possible selection bias. Furthermore, the unavailability of several variables in the included studies, such as data regarding functional capacity and the number of patients in different CP class was an additional limitation. Moreover, in some studies only the cirrhotic patients on the waiting list for LT were included. Finally, the included studies used different methods and cut-offs (without considering important variables such as ethnicity) to define myosteatosis, since there are no well-established and universally accepted criteria for the diagnosis of myosteatosis. However, eleven of the 17 included studies used the BMI-based definition and the same cut-offs, while we preformed separate analyses focusing on studies that used BMI- and gender-based criteria, whenever possible. Nevertheless, our meta-analysis is the first one dealing with this topic, while several sub-group analyses were performed with clinically useful findings.

In conclusion, the present analysis indicated the high prevalence of myosteatosis among the patients with end stage liver disease, particularly in those with NAFLD-associated cirrhosis. However, no difference in the prevalence of myosteatosis was found based on gender and race of the patients. As may be expected, patients with myosteatosis, compared to those without, had more frequently T2DM, while the association between myosteatosis and hepatic encephalopathy was also confirmed. Interestingly, myosteatosis seems to have a negative impact on the outcome of patients with chronic liver disease, which is very important in daily clinical practice for the early detection and incorporation of myosteatosis in the protocol management of these patients (including dietary measurements, physical exercise, medication) to avoid liver-related complications and to improve survival.

## Ethical guidelines statement

The study has been performed in accordance with the ethical standards laid down in the 1964 Declaration of Helsinki and its later amendments. The manuscript does not contain clinical studies or patient data.

## Informed consent in studies with human subjects and animal studies

Not applicable.

### Supplementary Information

Below is the link to the electronic supplementary material.

PRISMA flow diagram of study selection (TIF 68 KB)

Forest plot of studies comparing the prevalence of myosteatosis according to the gender (males vs females) (TIF 466 KB)

Forest plot of studies comparing the prevalence of myosteatosis in patients with cirrhosis and in patients with chronic liver disease (TIF 477 KB)

Funnel plot of the meta-analysis of included studies (TIF 67 KB)

Supplementary file5 (DOCX 21 KB)

Supplementary file6 (DOCX 16 KB)

## Data Availability

The data presented in this study are available on request from the corresponding author.

## Abbreviations

CT: Computed tomography
MRI: Magnetic resonance imaging
L3: Third lumbar vertebra
LT: Liver transplantation
NAFLD: Non-alcoholic fatty liver disease
ALD: Alcoholic liver disease
CP: Child-Pugh
MELD: Model for End-Stage Liver Disease
HCC: Hepatocellular carcinoma
T2DM: Type II diabetes mellitus
The NOS: Newcastle-Ottawa scale
BMI: Body mass index
CI: Confidence interval
