# Supplementary figures and images for: Prevalence and impact on the outcome of myosteatosis in patients with cirrhosis: a systematic review and meta-analysis
Source: Hepatol Int. 2024 Feb 8;18(2):688–99. doi: 10.1007/s12072-023-10632-8 (PMC11014812; doi:10.1007/s12072-023-10632-8)

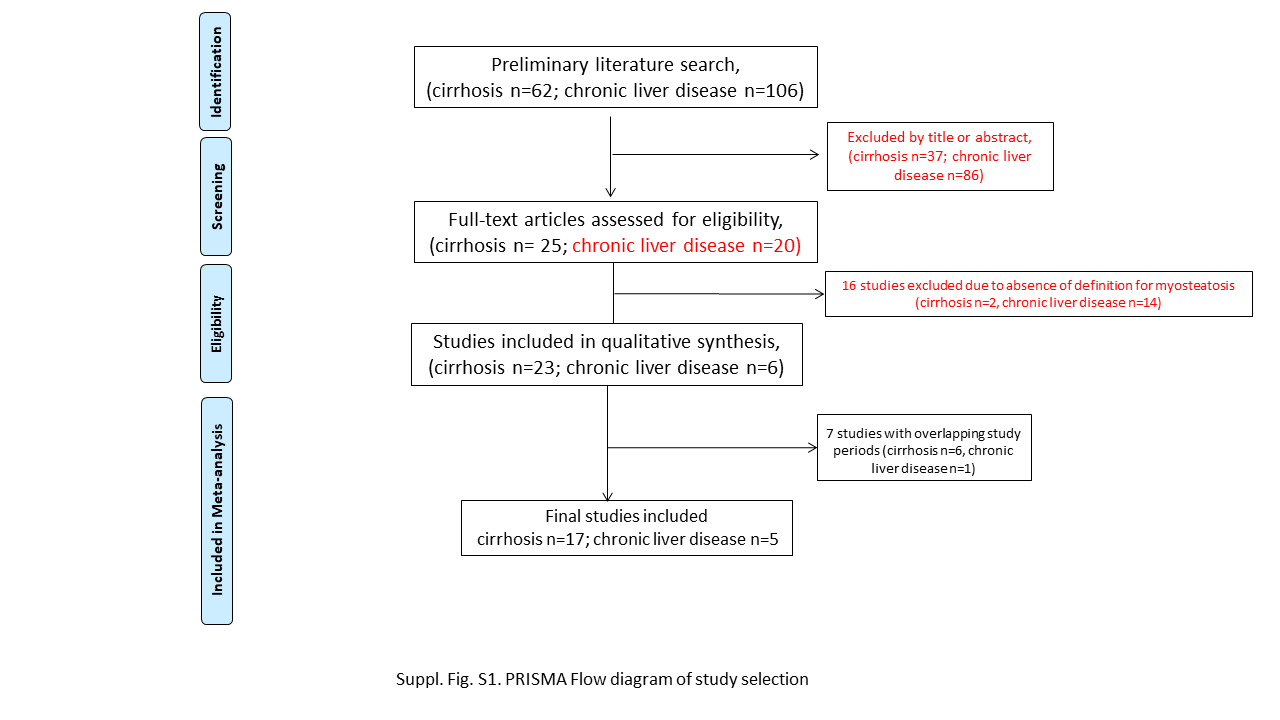

Supplement: Supplementary file 1 — PRISMA flow diagram of study selection (TIF 68 KB) [file 12072_2023_10632_MOESM1_ESM.tif]

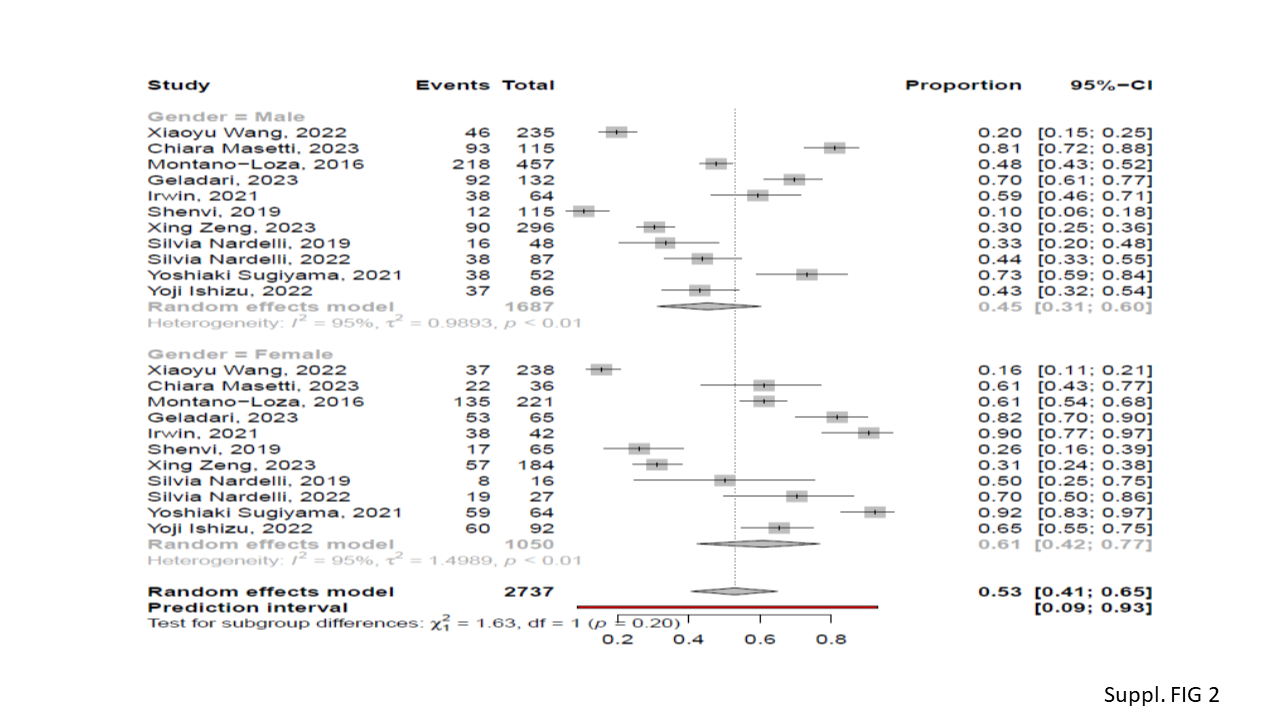

Supplement: Supplementary file 2 — Forest plot of studies comparing the prevalence of myosteatosis according to the gender (males vs females) (TIF 466 KB) [file 12072_2023_10632_MOESM2_ESM.tif]

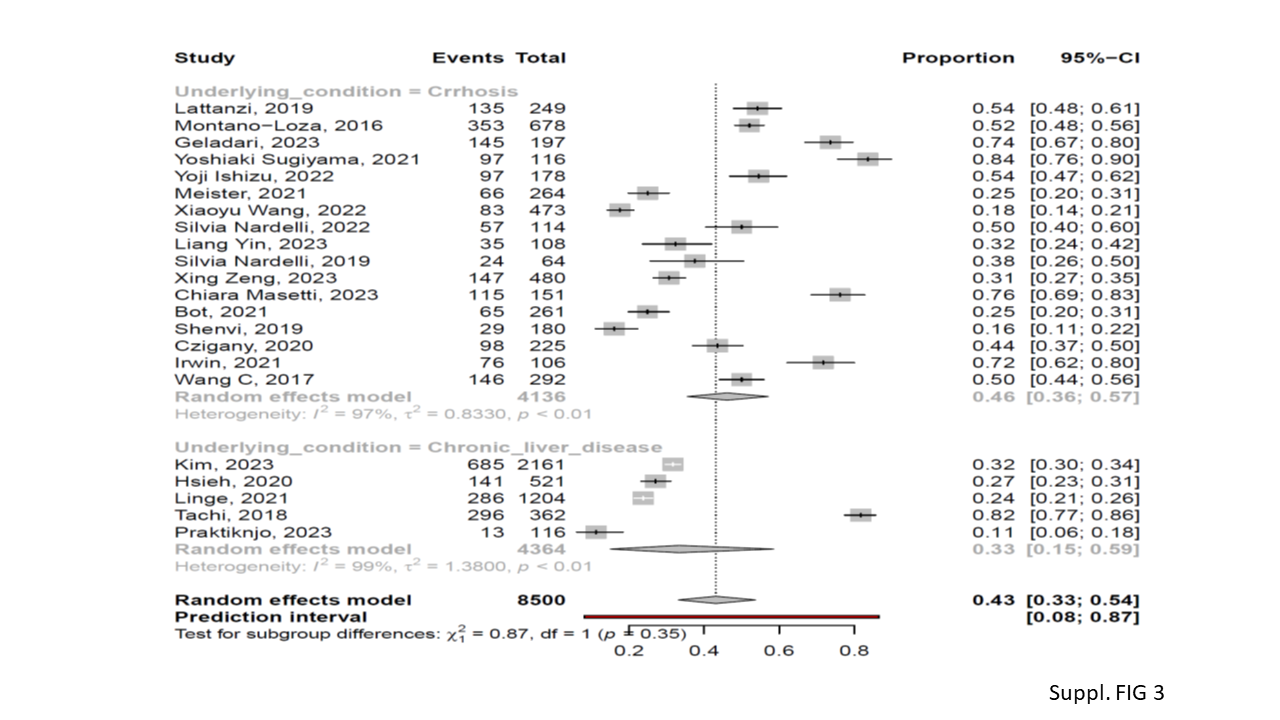

Supplement: Supplementary file 3 — Forest plot of studies comparing the prevalence of myosteatosis in patients with cirrhosis and in patients with chronic liver disease (TIF 477 KB) [file 12072_2023_10632_MOESM3_ESM.tif]

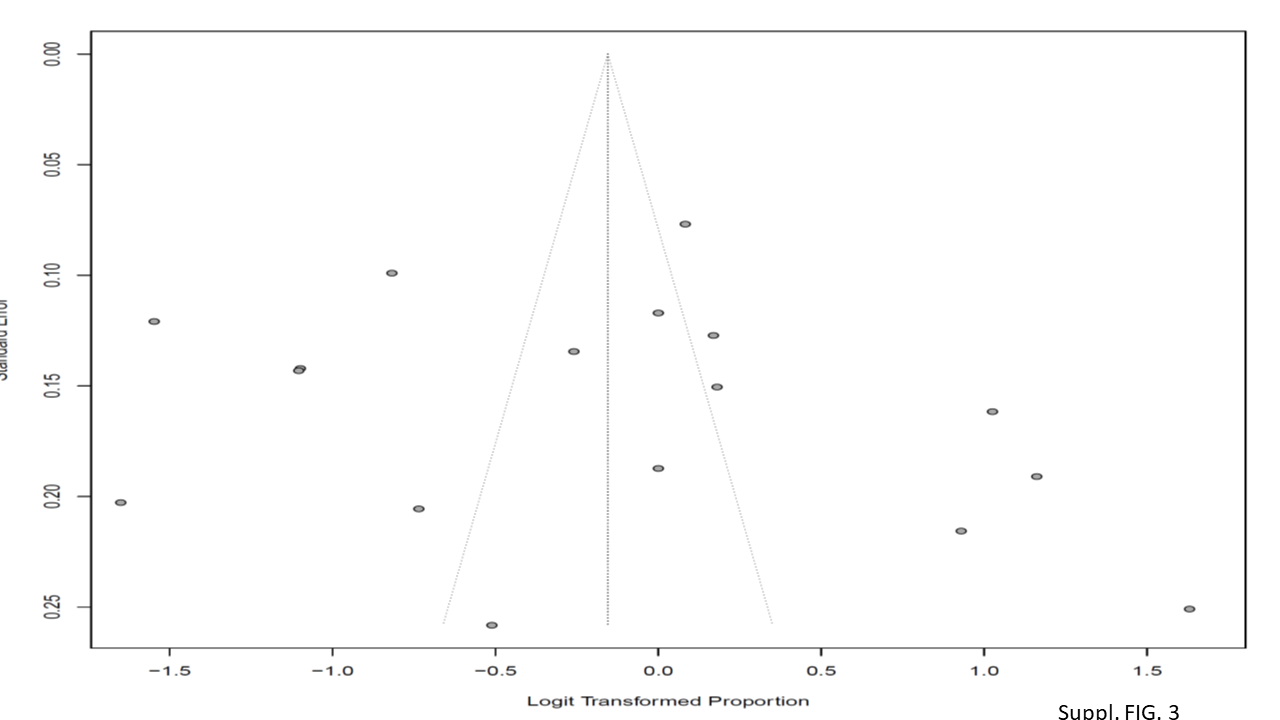

Supplement: Supplementary file 4 — Funnel plot of the meta-analysis of included studies (TIF 67 KB) [file 12072_2023_10632_MOESM4_ESM.tif]
