# Supplementary material for: Prevalence and impact on the outcome of myosteatosis in patients with cirrhosis: a systematic review and meta-analysis
Source: Hepatol Int. 2024 Feb 8;18(2):688–99. doi: 10.1007/s12072-023-10632-8 (PMC11014812; doi:10.1007/s12072-023-10632-8)
Supplement: Supplementary file 5 — Supplementary file5 (DOCX 21 KB) [file 12072_2023_10632_MOESM5_ESM.docx]

**Table 1.** Published studies regarding the characteristics of cirrhotic patients with myosteatosis, compared to those without myosteatosis**.**

| **First author, Country, Publication year, Study design (Ref.)** | **Newcastle-Ottawa Scale** | **Definition of myosteatosis** | **Number of patients**  **n/n** | **Male sex,**  **n/n** | **Alcohol n/n** | **NAFLD n/n** | **Viral hepatitis n/n** | **Diabetes mellitus, n/n** | **History of encephalopathy,**  **n/n** | **CP class A, B, C**  **n/n** | **Death, n/n,**  **(causes of death)** |
| --- | --- | --- | --- | --- | --- | --- | --- | --- | --- | --- | --- |
| Lattanzi, Italy, 2019, RS [11] | 8 | BMI-based, | 135/114 | NA/NA | 35/25 | 4/4 | 77/65 | NA/NA | 69/35 | 33,58,44/54, 41,19 | 48/17, (NA) |
| Montano-Loza, Canada, 2016, PS [12] | 8 | BMI-based | 353/325 | 218/239 | 86/67 | 88/8 | 145/167 | 55/NA | NA/NA | 43, 180, 130/ NA | 145/114, (NA)* |
| Geladari, Greece, 2023, PS [13] | 7 | BMI-based | 145/52 | 92/40 | 65/20 | NA/NA | 27/19 | NA/NA | 37/4 | NA/NA | 40/0,  (all liver-related) |
| Yoshiaki Sugiyama, Japan, 2021, RS [14] | 6 | BMI-based | 97/19 | 38/14 | 11/3 | NA/NA | 51/11 | 34/9 | NA/NA | 71,22,4/12,7,0 | NA/NA, (NA) |
| Yoji Ishizu, Japan, 2022, RS [15] | 6 | BMI-based | 97/81 | 37/55 | 17/14 | NA/NA | 44/49 | 19/23 | NA/NA | 75,21,1/67,13,1 | 14/9, (NA) |
| Meister, Germany, 2021, RS [16] | 8 | Gender-based | 66/198 | NA/NA | NA/NA | NA/NA | NA/NA | NA/NA | NA/NA | NA/NA | NA/NA, (NA) |
| Xiaoyu Wang, China, 2022, RS [17] | 7 | Gender-based | 83/390 | 46/189 | 15/85 | 25/104 | 20/117 | 19/63 | 13/41 | 16, 52, 15/  115, 236, 39 | NA/NA, (NA) |
| Silvia Nardelli, Italy, 2022, PS [18] | 8 | BMI-based | 57/57 | 38/49 | 6/7 | 21/11 | 24/38 | NA/NA | 33/14 | 14,33,10/18,32,7 | 20/5, (NA) |
| Liang Yin, China, 2023, RS [19] | 7 | BMI-based | 35/73 | NA/NA | NA/NA | NA/NA | NA/NA | NA/NA | NA/NA | NA/NA | NA/NA, (NA) |
| Silvia Nardelli, Italy, 2019, PS [20] | 8 | BMI-based | 24/40 | 16/32 | 6/7 | 1/5 | 17/28 | NA/NA | 10/10 | 6,13,5/14,20,6 | 20/5, (NA) |
| Xin Zeng, China, 2023, PS [21] | 9 | Gender-based | 147/333 | 90/206 | 24/37 | 33/65 | 45/168 | 44/52 | 20/26 | NA/NA | NA/NA, (NA) |
| Chiara Masetti, Italy, 2023, RS [22] | 8 | Gender-based | 115/36 | 93/22 | 30/4 | 19/4 | 58/18 | 34/11 | NA/NA | 99, 15, 1/  25, 11, 0 | 65/20, (NA) |
| Bot, the Netherlands, 2021, RS [23] | 8 | other criteria | 65/196 | NA/NA | NA/NA | NA/NA | NA/NA | NA/NA | NA/NA | NA/NA | NA/NA, (NA) |
| Shenvi, USA, 2019, RS [24] | 7 | other criteria | 29/151 | 12/103 | 4/21 | 7/21 | 11/81 | NA/NA | NA/NA | NA/NA | NA/NA, (NA) |
| Czigany, Germany, 2020, PS [25] | 8 | BMI-based | 98/127 | NA/NA | NA/NA | NA/NA | NA/NA | NA/NA | NA/NA | NA/NA | NA/NA, (NA) |
| Irwin, South Africa, 2021, RS [26] | 8 | BMI-based | 76/30 | 38/NA | 19/NA | 5/NA | 2/4 | 15/4 | NA/NA | NA/NA | NA/NA, (NA) |
| Wang C, USA, 2017, PS [27] | 8 | BMI-based | 146/146 | NA/NA | NA/NA | NA/NA | NA/NA | NA/NA | NA/NA | NA/NA | NA/NA, (NA) |

NA not available; RS: retrospective study, PS: prospective study; BMI: body mass index; NAFLD: non alcoholic fatty liver disease; CP: Child-Pugh; mo: months.

All n/n results: number of patients with myosteatosis / number of patients without myosteatosis

* Patients with cirrhosis and myosteatosis, compared to those with myosteatosis, had more frequent sepsis-related deaths and less frequent liver-related deaths
