# Supplementary material for: Prevalence and impact on the outcome of myosteatosis in patients with cirrhosis: a systematic review and meta-analysis
Source: Hepatol Int. 2024 Feb 8;18(2):688–99. doi: 10.1007/s12072-023-10632-8 (PMC11014812; doi:10.1007/s12072-023-10632-8)
Supplement: Supplementary file 6 — Supplementary file6 (DOCX 16 KB) [file 12072_2023_10632_MOESM6_ESM.docx]

**Suppl. Table 2.** Published studies regarding the characteristics of patients with chronic liver disease with myosteatosis, compared to those without myosteatosis**.**

| **First author, Country, Publication year, (Ref.)** | **Total number, number with cirrhosis** | **Definition of myosteatosis** | **Number of patients**  **n/n** | **Male sex,**  **n/n** | **Alcohol n/n** | **NAFLD n/n** | **Viral hepatitis n/n** | **Other causes** | **Diabetes mellitus, n/n** | **Death, n/n,**  **(causes of death)** |
| --- | --- | --- | --- | --- | --- | --- | --- | --- | --- | --- |
| Kim, Korea, 2023, [35] | 2161/0 | Other criteria | 685/1476 | NA/NA | 0/0 | 685/1476 | 0/0 | 0/0 | NA/NA | NA/NA, (NA) |
| Hsieh, Taiwan, 2021, [36] | 521/NA | BMI-based | 141/380 | 24/241 | 0/0 | 141/380 | 0/0 | 0/0 | 78/127 | NA/NA, (NA) |
| Linge, Sweden, 2020, [37] | 1204/NA | Gender-based | 286/918 | NA/NA | 0/0 | 286/918 | 0/0 | 0/0 | NA/NA | NA/NA, (NA) |
| Tachi, Japan, 2018, [38] | 362/94 | Other criteria | 296/66 | 161/15 | NA/NA | NA/NA | NA/NA | NA/NA | NA/NA | NA/NA, (NA) |
| Praktiknjo, Germany, 2023, [39] | 116/NA | Other criteria | 13/103 | 7/67 | 0/0 | 0/0 | 0/0 | 13*/103* | NA/NA | NA/NA, (NA) |

NA not available; BMI: body mass index; NAFLD: non alcoholic fatty liver disease.

All n/n results: number of patients with myosteatosis / number of patients without myosteatosis

*All patients had primary sclerosing cholangitis
